# Association of dietary carbohydrate intake with bone mineral density, osteoporosis and fractures among adults without diabetes: Evidence from National Health and Nutrition Examination Survey

**DOI:** 10.1016/j.heliyon.2024.e35566

**Published:** 2024-08-02

**Authors:** Ran Chen, Kai Gong, Wei Chen, Zongfeng Chen, Lianyang Zhang, Ying Tang, Yang Li, Siru Zhou

**Affiliations:** a War Trauma Medical Center, State Key Laboratory of Trauma and Chemical Poisoning, Army Medical Center, Daping Hospital, Army Medical University, Chongqing, 400042, PR China; b Department of Orthopaedics, The First Affiliated Hospital of Chengdu Medical College, Chengdu, Sichuan Province, 610500, PR China

**Keywords:** Dietary carbohydrate intake, Bone mineral density, Osteoporosis, Fracture, NHANES

## Abstract

**Background:**

The impact of dietary carbohydrate intake on bone health remains a subject of controversy, potentially influenced by individuals with diabetic osteoporosis who exhibit normal or elevated bone mineral density (BMD). The cross-sectional study was conducted to explore the association between carbohydrate intake and BMD, osteoporosis and fractures among adults without diabetes, based on the National health and nutrition examination survey (NHANES).

**Methods:**

Participants were from the NHANES 2005–2010, excluding individuals with diabetes and those with incomplete data. The association between carbohydrate intake and BMD was analyzed using Spearman correlation, linear regression analysis and subgroup analysis, respectively. The association between carbohydrate intake and osteoporosis/fractures was analyzed using weighted logistic regression analysis.

**Results:**

A total of 7275 adult participants were included and their dietary carbohydrate intake was inversely associated with BMD in the total femur [β = −0.20 95%CI (−0.30, −0.10); p < 0.001], femoral neck [β = −0.10 95%CI (−0.20, −0.00); p = 0.002], and lumbar spine [β = −0.10 95%CI (−0.20, −0.00); p = 0.004]. Stratified analysis indicated that individuals aged 65 and over, women, and non-Hispanic whites were more likely to have lower BMD. Furthermore, a higher intake of dietary carbohydrates was associated with an increased risk of osteoporosis [OR = 1.001 95%CI (1.001, 1.001); p < 0.001] and fractures at the hip [OR = 1.005 95%CI (1.005, 1.005); p < 0.001], wrist [OR = 1.001 95%CI (1.001, 1.001), p < 0.001], and spine [OR = 1.003 95%CI(1.003, 1.003); p < 0.001].

**Conclusions:**

A higher carbohydrate diet is associated with lower BMD and a higher risk of osteoporosis and fractures among adults without diabetes, and a higher carbohydrate consumption show a stronger effect in individuals aged 65 and over, women, and non-Hispanic whites.

## 1 Introduction

Osteoporosis, one of the most prevalent and severe systemic metabolic bone diseases, is characterized by a decline in bone mineral density (BMD), bone strength, and degeneration of bone microstructure [1]. In recent years, the aging population and increasing life expectancy have contributed to a steady rise in osteoporosis incidence worldwide. Currently, an estimated 200 million individuals globally are affected by osteoporosis, with projected increases of 310 % in male and 240 % in female osteoporosis prevalence by 2050 [2]. Osteoporosis patients with diminished bone strength and heightened fragility are susceptible to fractures resulting from low-energy trauma. Consequently, there is a concomitant increase in fragility fractures at the hip, spine, and wrist [3], leading to increased mortality risk, prolonged decline in physical function and decreased quality of life [4]. Hence, it is of paramount importance to explore effective methods for early prediction, diagnosis, and intervention of osteoporosis.

BMD is a crucial indicator for evaluating skeletal health, and predicting the risks of osteoporosis and fragility fractures. Typically, bone mineral mass peaks during adolescence and gradually declines thereafter [5]. Various factors, including dietary intake, can influence this process. Protein, fat and carbohydrates are the three primary macronutrients in daily diets. In recent years, extensive research has examined the association between these macronutrients and bone metabolism. For instance, protein intake has demonstrated positive associations with BMD, and higher protein intakes may mitigate fracture risk [6]. Conversely, individuals with a high-fat diets are more susceptible to osteoporosis, as such diets are associated with decreased BMD and accelerated bone loss [7]. With regard to carbohydrates, previous studies have suggested that increased consumption of certain carbohydrates, such as processed and snack foods, may be linked to lower BMD [8,9]. Moreover, recent findings have indicated that dietary patterns characterized by a higher proportion of carbohydrate could be detrimental to bone health and elevate the risk of bone loss [10]. Nevertheless, the impact of carbohydrate intake on fracture risk remains controversial. Systematic reviews encompassing multiple cohort studies failed to observe a significant association between carbohydrate intake and fracture risk [[11], [12], [13], [14], [15]], whereas several studies conducted in the United States have found that increased carbohydrate intake can significantly reduce the risk of fractures in older adults [16,17].

These inconsistent results in previous studies may be attributed at least in part to the limited attention given to patients with diabetic osteoporosis. Unlike classical osteoporosis, individuals with diabetic osteoporosis often exhibit normal or increased BMD [[18], [19], [20], [21]]. Therefore, restricting the study population to non-diabetic individuals would contribute to a better understanding of the effect of carbohydrate intake on bone health. Additionally, blood glucose levels are found to be positively associated with BMD and for every 1 mmol/L increase in blood glucose, BMD increases by 4.64–6.88 mg/cm^2^, consequently reducing the risk of fractures [[22], [23], [24], [25]]. Carbohydrates are the macronutrients with the strongest impact on raising blood glucose levels, it raises the question of whether increasing carbohydrate intake in non-diabetic individuals could ultimately improve bone density and reduce the risk of fractures? It is essential to clarify this point, especially for professional athletes and enthusiasts participating in endurance races. Their rigorous training regimes and intense competitive events necessitate a high carbohydrate intake [26,27]. However, if this carbohydrate consumption adversely affects bone health, it undoubtedly escalates their risk of bone fractures and related health hazards. To the best of our knowledge, no comprehensive research addressing this question has been conducted thus far. To fill this gap, the study analyzed the association between dietary carbohydrate intake and bone health in a non-diabetic population based on data from the National Health and Nutrition Survey (NHANES).

## 2 Methods

### 2.1 Study population

This cross-sectional study aimed to explore the potential association between dietary carbohydrate intake and BMD as well as the risk of osteoporosis and fractures among adults in the US. Data from NHANES (2005–2010) was used because the three cycles also included continuous recording of data of interest, such as BMD at the total hip, femoral neck, and lumbar spine, osteoporosis, and fracture questionnaires. NHANES is a series of continuous surveys conducted by the National Center for Health Statistics (NCHS) at the Centers for Disease Control and Prevention (CDC). Initiated approximately sixty years ago, NHANES aims to assess the health and nutritional status of diverse populations in the United States, while also investigating various health concerns. NHANES is conducted annually, selecting a sample population of five thousand individuals from multiple counties across the country, with fifteen counties visited each year. Ethical approval for all NHANES protocols was obtained from the NCHS Ethics Review Board (NHANES2009-2010: Continuation of Protocol #2005–06; NHANES2007-2008: Continuation of Protocol #2005–06; NHANES2005-2006: Protocol #2005–06). Participation in the survey are required to provide informed consent, along with comprehensive demographic information, examination data, laboratory test results, and questionnaire responses for research purposes.

**Fig. 1:**
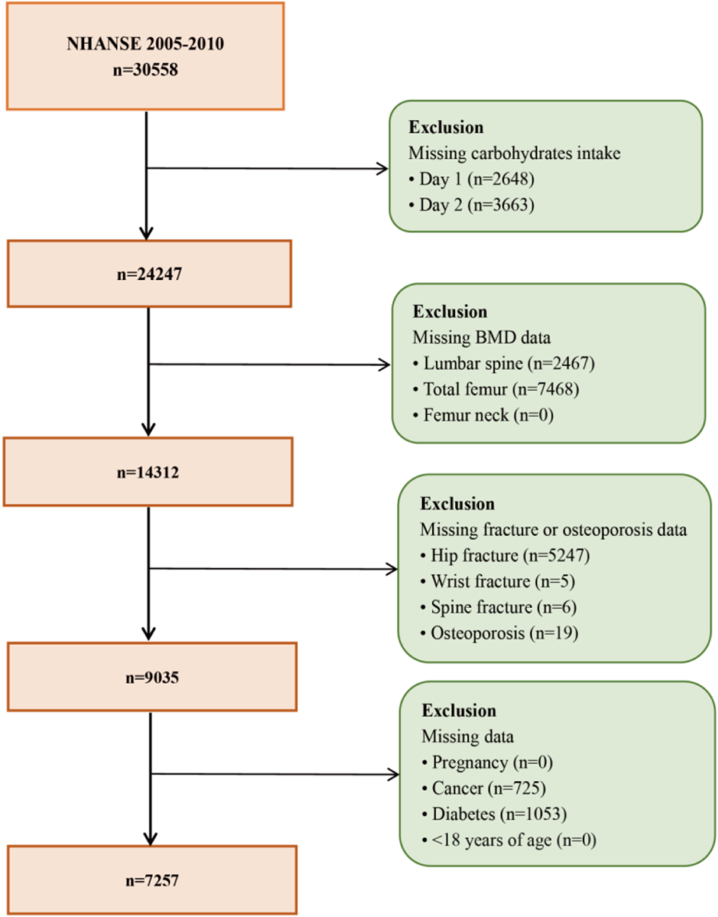
Flowchart displaying selection criteria of the study population.

The present study utilized data from three cycles of the NHANES survey conducted between 2005 and 2010. The study population consisted of 30,588 individuals who provided comprehensive demographic information, completed health questionnaires, underwent laboratory tests, and had their BMD measured and medical history of osteoporosis and fractures recorded. To ensure data reliability, specific exclusion criteria was applied (Fig. 1). Firstly, the study excluded 6331 individuals due to missing dietary carbohydrate intake data. Secondly, the study excluded 9935 participants who had missing data on BMD. Thirdly, the study excluded 5277 participants with missing data on osteoporosis and fracture. Additionally, the study excluded 1778 participants who had type 1 or 2 diabetes, cancer, pregnancy, or were under 18 years old. Consequently, the final analysis included a total of 7257 individuals.

### 2.2 BMD measurement and osteoporosis diagnosis

Dual-energy X-ray absorptiometry (DXA) in NHANES was employed as the primary method for assessing BMD in the years 2005–2010. DXA is widely recognized for its reliability and accuracy in measuring BMD. The specific DXA equipment used in this study was the Hologic QDR 4500A fan beam densitometer, manufactured by Hologic Inc., located in Bedford, MA, USA. To evaluate the BMD levels, measurements were taken at different anatomical sites, including the lumbar spine and total femur area. For the lumbar spine, the BMD level was estimated as the mean value of the first to fourth lumbar vertebrae. This approach provided a comprehensive assessment of BMD in the lumbar region, known to be a critical area for osteoporosis evaluation. The evaluation of the proximal femur regions focused primarily on the left hip. However, if there was evidence of a left-hip replacement or the presence of metal items in the left leg, the right hip was evaluated instead. This decision ensured that accurate BMD measurements could be obtained without interference from previous surgeries or metal implants [28]. Radiological technologists who were licensed and trained performed all DXA examinations. Rigorous training and adherence to standardized protocols were followed to maintain consistency and reliability throughout the data collection process. The NHANES survey and DXA scans invited individuals aged 8 and above to participate. However, specific exclusion criteria were applied to ensure the integrity and validity of the data. Individuals with bilateral hip fractures, replacements or pins in both hips, a body weight exceeding 300 pounds, ongoing pregnancy (confirmed through a positive urine pregnancy test or self-report), or recent involvement in nuclear medicine studies within the past three days were excluded from the DXA examinations. These criteria aimed to eliminate potential confounding factors that could affect the accuracy of BMD measurements and ensure a more homogeneous and representative study population [29].

In this study, the femoral neck bone density of participants was transformed into T-scores using the formula: T-score = (participant's bone density - mean bone density of the reference group)/standard deviation (SD) of the reference group's bone density [30]. In line with previous studies, osteoporosis in the included participants was defined as meeting one of the following criteria: (1) Physician diagnosis; (2) Current usage of anti-osteoporotic medications; (3) Femoral neck T-score ≤ −2.5 [[30], [31], [32]].

### 2.3 Dietary carbohydrate intake

The nutritional intake data, including carbohydrates and other nutrients, for all participants was documented during the dietary interview. This information comprised the types and amounts of food and beverages (including all types of water) consumed by respondents within the 24 h preceding the interview, spanning from midnight to midnight. Specific details outlining the primary dietary constituents consumed by respondents are detailed in individual food files within the NHANES database. In the present study, the dependent variable was dietary carbohydrates and dietary carbohydrates from foods was calculated using the US Department of Agriculture's Food and Nutrient Database for Dietary Studies, which provided detailed information about the main food components and the amounts of carbohydrates obtained from each food. Each participant provided intake data for two non-consecutive days between the years 2005 and 2010. Data for the first day was collected at a mobile examination center, while the data for the second day was collected through telephone interviews conducted 3–10 days later. For this analysis, the average carbohydrates from the two non-consecutive days were considered as the measure of dietary carbohydrate intake. Given the skewed distribution of dietary carbohydrate intake among the study population, the study categorized the data into tertiles: the lowest tertile (T1, <206.29 g/day), the middle tertile (T2, 206.29–291.56 g/day), and the highest tertile (T3, >291.56 g/day).

### 2.4 Covariates

According to previous reports [2,10,11,16,17] and the analysis in Table 1, an array of potential confounding variables from the comprehensive collection of demographics, examination, laboratory, and questionnaire data were identified. These covariates encompass the following factors: age (continuous), gender (male, female), race/ethnicity (non-Hispanic white, non-Hispanic black, Mexican American, others), education levels (below high school, high school, above high school), poverty income ratio (PIR), body mass index (BMI) (kg/m^2^), physical activity level (low, moderate, vigorous), alcohol consumption patterns (non-drinking, moderate drinking, heavy drinking), smoking (non-smoking, passive smoking, active smoking), arthritis (yes, no), hypertension (yes, no), coronary heart disease (yes, no), stroke (yes, no), thyroid problem (yes, no), albumin levels (g/dL), total protein (ng/dL), total calcium (mmol/L), serum vitamin D (nmol/L), phosphorus(mmol/L), alkaline phosphatase(U/L), triglycerides (mmol/L), total cholesterol (mmol/L), blood urea nitrogen, uric acid (mmol/L), serum glucose (mmol/L), bilirubin (mg/dL), the intake of calcium, phosphorus, total cholesterol, total energy, fat, protein. The classification of some covariates was detailed in Table S1. After univariate logistics regression analysis (Table S2) and collinearity diagnosis, the study excluded variables with statistical significance: p > 0.05 or variance inflation factor (VIF) > 10: the intake of phosphorus, total energy, fat, and protein, and the remaining variables were used for further analysis.

**Table 1:** Baseline characteristics of study participants in NHANES 2005–2010.

	Total participant, N = 7257		
Variable	Osteoporosis n = 271	Non-osteoporosis n = 6986	p value
Age (years)	64 (53, 70)	41 (30 51)	<0.001
Gender			<0.001
Men	20 (7.43 %)	3565 (51.03 %)	
Women	251 (92.57 %)	3421 (48.97 %)	
Race/ethnicity			<0.001
Non-Hispanic white	230 (84.82 %)	4853 (69.47 %)	
Non-Hispanic black	14 (5.09 %)	766 (10.96 %)	
Mexican American	10 (3.54 %)	635 (9.09 %)	
Others	17 (7.10 %)	732 (10.48 %)	
Education levels			0.003
Under high school	75 (22.40 %)	1702 (15.75 %)	
High school	81 (30.99 %)	1606 (22.90 %)	
Above high school	115 (46.61 %)	3678 (61.35 %)	
PIR			0.083
Low income	41 (11.11 %)	1248 (12.21 %)	
Middle income	170 (59.43 %)	3904 (50.73 %)	
High income	60 (29.46 %)	1834 (37.06 %)	
BMI			0.021
Normal	111 (44.71 %)	2343 (36.26 %)	
Overweight	97 (34.68 %)	2592 (35.90 %)	
Obese	63 (20.61 %)	2051 (27.84 %)	
Waist circumference (cm)	91.8 (82.6, 100.5)	94.4 (84, 103)	<0.001
Physical activity			0.031
Low	137 (44.07 %)	2648 (34.58 %)	
Moderate	14 (4.45 %)	339 (5.06 %)	
High	49 (16.32 %)	1175 (26.28 %)	
Missing	71 (35.10 %)	2124 (34.09 %)	
Alcohol drinking			<0.001
Non-drinking	57 (14.89 %)	752 (8.99 %)	
Moderate drinking	142 (58.08 %)	2976 (43.54 %)	
Heavy drinking	72 (27.03 %)	3258 (47.47 %)	
Smoking			0.001
Non-smoking	70 (24.74 %)	1260 (18.55 %)	
Passive smoking	147 (54.18 %)	3853 (54.62 %)	
Active smoking	54 (21.08 %)	1873 (26.83 %)	
Hypertension			<0.001
Yes	106 (38.39 %)	1725 (22.50 %)	
No	165 (61.61 %)	5261 (77.50 %)	
Arthritis			<0.001
Yes	164 (58.86 %)	1178 (15.79 %)	
No	107 (41.14 %)	5808 (84.21 %)	
Coronary heat disease			<0.001
Yes	14 (4 %)	127 (1.29 %)	
No	257 (96 %)	6859 (98.71 %)	
Stroke			<0.001
Yes	18 (7.11 %)	106 (1.24 %)	
No	253 (92.89 %)	6880 (98.76 %)	
Albumin (g/dL)	42 (40, 44)	43 (41, 45)	<0.001
Total calcium (mmol/L)	2.350 (2.30, 2.45)	2.450 (2.325, 2.475)	<0.001
Vitamin D (nmol/L)	66.5 (53.5, 82.9)	61.24 (51, 78.6)	<0.001
Phosphorus (mmol/L)	1.259 (1.162, 1.388)	1.21 (1.098, 1.324)	<0.001
ALP (U/L)	67 (1.098, 1.324)	66 (52, 75)	<0.001
Triglycerides (mmol/L)	1.332 (0.982, 2.156)	1.344 (0.892, 1.998)	<0.001
Total cholesterol (mmol/L)	5.146 (4.422, 5.974)	5.069 (4.422, 5.741)	<0.001
BUN (mmol/L)	4.64 (3.93, 5.71)	4.28 (3.57, 5.00)	<0.001
Uric acid (mmol/L)	279.6 (232.0, 321.2)	315.2 (261.7, 368.8)	<0.001
ALT (U/L)	20 (15, 26)	22 (17, 28)	<0.001
AST (U/L)	24 (20, 27)	25 (20, 27)	<0.001
Bilirubin (mg/dL)	0.7 (0.5, 0.8)	0.8 (0.6, 0.9)	<0.001
Calcium intake (mg)	720 (565, 999)	851.5 (623.5, 1220.5)	<0.001
Phosphorus intake (mg)	1066 (821, 1362)	1271.5 (978.5, 1695)	<0.001
Total cholesterol intake (mg)	196.5 (137, 335)	243.5 (157.5, 371.5)	<0.001
Total energy intake (Kcal)	1653.50 (1347.5, 2195.5)	2010.25 (1573, 2665)	<0.001
Fat intake (g)	61.56 (47.07, 81.57)	72.67 (54.72, 102.69)	<0.001
Protein intake (g)	63.72 (49.14, 78.95)	77.46 (60.17, 104.17)	<0.001
Carbohydrates intake (g)	215.28 (175.3, 571.5)	249.37 (187.93, 324.53)	<0.001

The continuous variables, which did not follow a normal distribution, were expressed by the median (IQR).

and the statistical significance was assessed using the Kruskal-Wallis test and Mann-Whitney *U* test; For categorical variables, the distribution was presented as N (%) and the statistical significance was assessed using the chi-square test. A two-sided p-value <0.05 was considered statistically significant. **Abbreviation:** BMI, body mass index; BMD, bone mineral density; ALP, Alkaline phosphatase; ALT, Alanine aminotransferase; AST, Alanine aminotransferase; BUN, Blood urea nitrogen. PIR, poverty income ratio; NHANES, National Health and Nutrition Examination Survey.

### 2.5 Statistical analysis

During the statistical analysis, the study incorporated the complex survey design elements of NHANES, including weighting, clustering, and stratification, in accordance with the NCHS normative analysis guidelines. In addressing sampling probability and nonresponse, the study employed the average dietary sampling weights over two consecutive days, divided by 3 (utilizing data from three NHANES cycles) to represent the noninstitutionalized population in the United States. The participants were initially divided into two groups based on their diagnosis of osteoporosis. The study described and analyzed the participants’ basic characteristics: The normality of the continuous variables was examined using the Kolmogorov-Smirnov test, and normally distributed continuous variables were described using means and standard deviations and analyzed using t-tests. Non-normally distributed continuous variables were presented as medians (Interquartile range, IQR) and analyzed using Kruskal-Wallis test and Mann-Whitney *U* test. Categorical variables were reported as percentages and analyzed using appropriate statistical tests, such as chi-square test. The Spearman correlation coefficient was used to assess the relationship between continuous variables, such as carbohydrate intake and BMD of total femur, femoral neck and lumbar spine, assuming an abnormal distribution. To assess the statistical differences among groups based on BMD levels at total femur, femoral neck and lumbar spine, a linear regression analysis was performed, adjusting for potential confounding factors to examine the relationship between carbohydrate intake levels and BMD. And these adjusted variables including age, gender, race/ethnicity, education levels, poverty income ratio, body mass index, smoking, physical activity level, alcohol consumption patterns, coronary heart disease, hypertension, albumin levels, total calcium, serum vitamin D, phosphorus, alkaline phosphatase, AST, ALT, triglycerides, total cholesterol, blood urea nitrogen, uric acid, serum glucose, bilirubin, total protein, arthritis, thyroid problem, stroke, the intake of calcium and total cholesterol. The effect size β and its 95%CI were calculated. In this study, a subgroup analysis based on age, gender, race and BMI was performed, with interactions tested. The variable of interest, carbohydrate intake, was categorized into three tertiles. The P-value for trend was calculated using the mean values of carbohydrate intake tertiles treated as a continuous variable. Weighted logistic regression was used with carbohydrate intake as the exposure and osteoporosis and fracture at hip, wrist and spine as the outcome to test the cross-tertiles trend test. For covariates with missing values, median interpolation was used for continuous variables, while an additional category was added for classified variables to evaluate the impact of missingness on the outcome. Data analyses were conducted using R (Version 4.3.1,2023-06-16 ucrt), http://www.Rproject.org), Graphpad prism 9.0 (https://www.graphpad-prism.cn) and EmpowerStats (http://www.empowerstats.com). Statistical significance was defined as a P-value below 0.05.

## 3 Results

### 3.1 Baseline characteristics of study participants

The baseline characteristics of the participants are presented in Table 1. A total of 7275 participants aged 18 years or older were included in this study. The median age of osteoporosis patients was 64 years, significantly higher than non-osteoporosis group (41 years). Despite having similar income levels, significant differences were observed between the two groups in multiple variables. It was found that participants who were non-Hispanic white, college-educated or above, physically inactive, moderate alcohol consumers, non-smokers, and had a history of hypertension, coronary heart disease, stroke, and arthritis were at a higher risk of osteoporosis. Moreover, participants with osteoporosis had lower levels of serum albumin, triglycerides, uric acid, AST, ALT, and bilirubin, while exhibiting higher levels of serum total calcium, vitamin D, phosphorus, alkaline phosphatase, total cholesterol, and BUN.

**Fig. 2:**
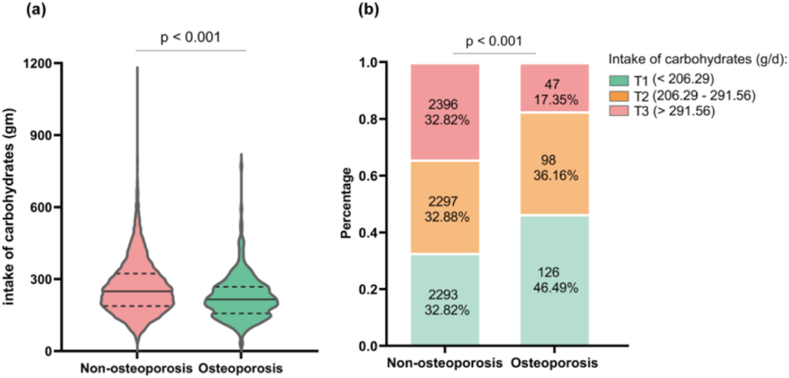
**Dietary carbohydrate intake level in osteoporosis and non-osteoporosis group. (a)** Dietary carbohydrate intake level by osteoporosis status (Mann-Whitney *U* test). **(b)** Distribution of dietary carbohydrate intake by osteoporosis status (Chi-square test).

Regarding dietary intake, osteoporosis group had significantly lower consumption of total energy, protein, fat, calcium, phosphorus, and total cholesterol compared to non-osteoporosis group. Notably, there was a significant difference in dietary carbohydrate intake between the osteoporosis group (215.28g/day) and the non-osteoporosis group (249.37g/day), indicating that there was a significantly lower carbohydrate intake among individuals with osteoporosis than non-osteoporosis group (p < 0.001) (Fig. 2a). Specifically, the study categorized dietary carbohydrate intake into three tertiles (the lowest tertile (T1, <206.29 g/day), the middle tertile (T2, 206.29–291.56 g/day), and the highest tertile (T3, >291.56 g/day)) to assess the BMD of the total hip, femoral neck, and lumbar spine among all participants. Further analysis revealed distinct distribution patterns of dietary carbohydrate intake between osteoporosis and non-osteoporosis groups (p < 0.001), and there was a significantly lower proportion of dietary carbohydrate intake in the highest tertile among individuals with osteoporosis (Fig. 2b).

### 3.2 Association of dietary carbohydrate intake with BMD

**Fig. 3:**
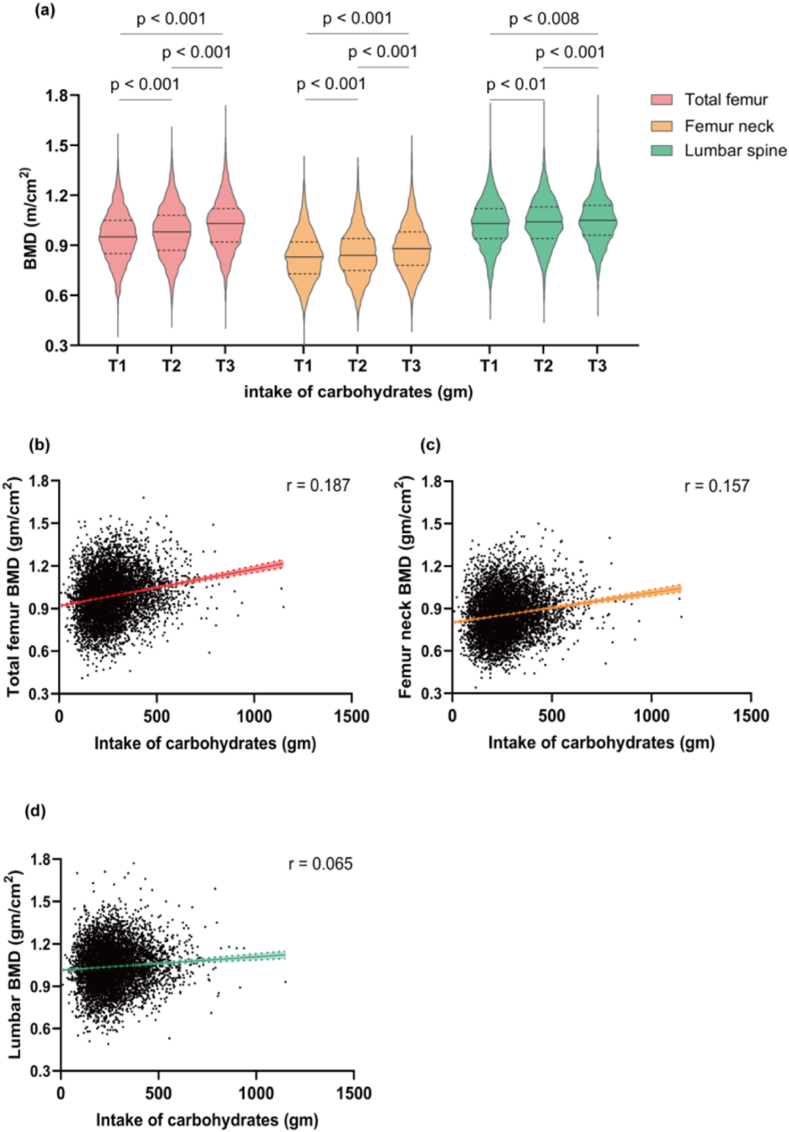
**The distribution and association between dietary carbohydrate intake and bone mineral density from NHANES 2005–2010**. **(a)** Distribution of bone mineral density at total femur, femoral neck and lumbar spine by dietary carbohydrate intake (Kruskal-Wallis test). **(b**–**d)** The association between dietary carbohydrate intake and bone mineral density of total femur, femoral neck and lumbar spine (Spearman analysis). Each green point represents a sample. Red solid rad line represents the smooth curve fit between variables. Red dashed lines represent the 95 % of confidence interval.

**Fig. 4:**
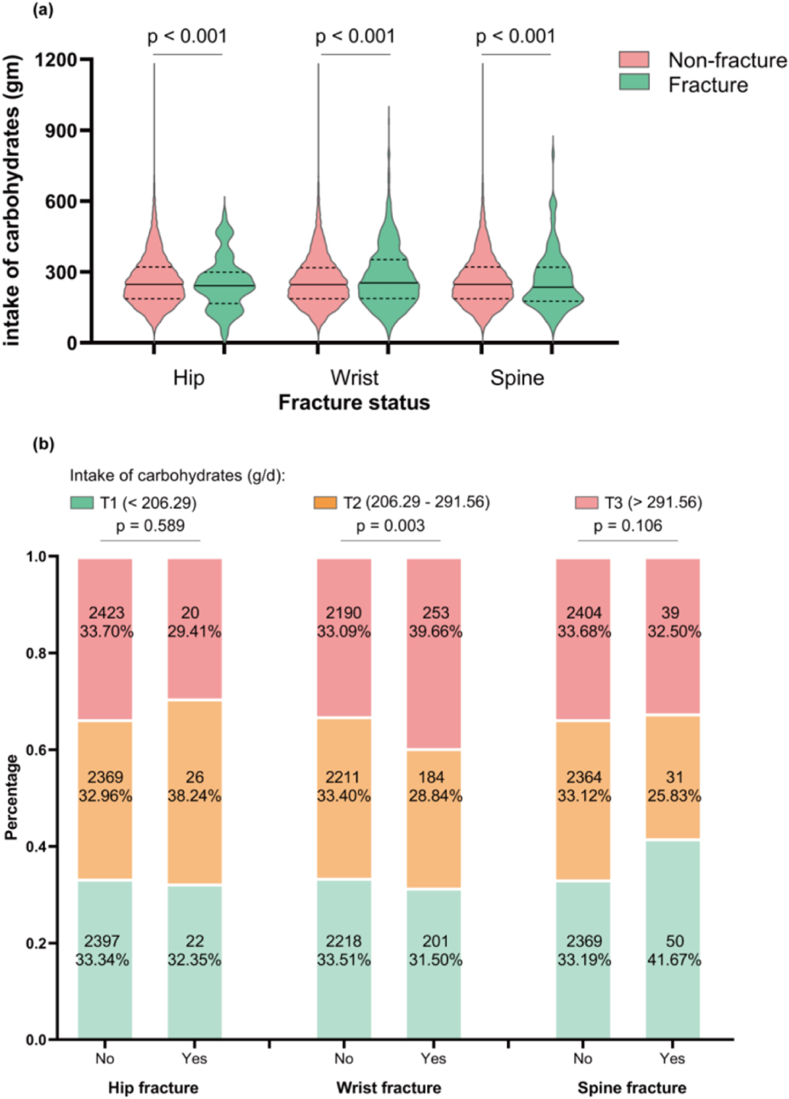
**Forest plot for the association of carbohydrate intake with bone mineral density of total femur, femoral neck and lumbar spine from NHANES 2005**–**2010**. Weighted multiple linear regression adjusted for age, gender, race/ethnicity, education levels, poverty income ratio, body mass index, smoking, physical activity level, alcohol consumption patterns, coronary heart disease, hypertension, albumin levels, total calcium, serum vitamin D, phosphorus, alkaline phosphatase, AST, ALT, triglycerides, total cholesterol, blood urea nitrogen, uric acid, serum glucose, bilirubin, total protein, arthritis, thyroid problem, stroke, the intake of calcium and total cholesterol. **Abbreviations**: CI, confidence interval; NHANES, National Health and Nutrition Examination Survey; BMD, bone mineral density; ALP, Alkaline phosphatase; ALT, Alanine aminotransferase; AST, Alanine aminotransferase; BUN, Blood urea nitrogen.

The observed disparity in carbohydrate intake between individuals with osteoporosis and non-osteoporosis participants prompted us to hypothesize a potential association between carbohydrate intake and BMD. To verify the hypothesis, the study first analyzed the BMD of the regions of interest (total femur, femoral neck, and lumbar spine) in different three tertiles of carbohydrate intake. The data of the study showed that BMD in the total hip, femoral neck, and lumbar spine all increased in accordance with higher carbohydrate intake (all p < 0.01), suggesting a potential positive association between carbohydrate intake and BMD (Fig. 3a). This association was further confirmed in Spearman correlation analysis, and the results indicated a significant positive association between carbohydrate intake and BMD of the total femur (r = 0.187, p < 0.001), femoral neck (r = 0.157, p < 0.001), and lumbar spine (r = 0.065, p < 0.001) (Fig. 3b–d). However, these positive results do not preclude the impact of relevant confounding factors on BMD. As is well known that BMD is influenced by various factors, including age, gender, race/ethnicity, dietary intake, and health status [5], so it is imperative to perform linear regression and control these factors to obtain a better understanding of the association between carbohydrate intake and BMD. After adjusting for relevant variables (Fig. 4), the results in weighted linear regression were contrary to previous association analysis, and revealed a significantly negative association between carbohydrate intake (as a continuous variable) and BMD in all regions of interest (total femur:[β, (95%CI), p-value: 0.20 (−0.30, −0.10), p < 0.001]; femoral neck: [β, (95%CI), p-value: 0.10 (−0.20, −0.00), p = 0.002]; lumbar spine: [β, (95%CI), p-value: 0.10 (−0.20, −0.00), p = 0.004]). This indicated that each 1g increase in carbohydrate intake was associated with a decrease of 0.1–0.2 mg/cm2 in BMD.

Furthermore, when considering carbohydrate intake as a categorical variable and comparing it with the lowest tertile, the study showed a significant inverse relationship between carbohydrate intake in the highest tertile and BMD. This was observed in the total femur [T, β, (95%CI), p-value: T3, -14.10 (−25.60, −2.50), p = 0.017], femoral neck: [T, β, (95%CI), p-value: T3, -11.10 (−21.10, −1.20), p = 0.036]; lumbar spine: [T, β, (95%CI), p-value: T3, -6.50 (−12.70, −0.50), p = 0.045]. Additionally, a trend test was conducted to evaluate the relationship between different levels of carbohydrate consumption and BMD. It was found that with the increase in carbohydrate intake, its negative effect on BMD was significantly enhanced: total femur (p-_trend_ = 0.034); femoral neck (p-_trend_ = 0.012); lumbar spine (p-_trend_ = 0.019).

### 3.3 The subgroup analyses between dietary carbohydrate intake and BMD stratifed by sex, age, race and BMI

To determine the relationship between carbohydrate intake and BMD across different subgroups, a stratified analysis was conducted based on sex, age, race, and BMI. The analysis, after adjusting for covariates, revealed that the impact of carbohydrate intake on BMD varies across diverse populations. Specifically, reduced BMD in the total femur was more likely to occur in individuals aged over 65 [T: β (95%CI): T3, -48.10 (−73.10, −23.10)], in women [T: β (95%CI): T3, -26.40 (−45.10, −7.70)] in non-Hispanic whites [T: β (95%CI): T3, -15.80 (−29.70, −1.90)](Fig. S1). Similarly, lower BMD in the femoral neck was also observed in participants with age over 65 [T: β (95%CI): T3, -48.60 (−72.40, −24.90)], in women [T: β (95%CI): T3, -30.20 (−48.40, −12.10)] in non-Hispanic whites [T: β (95%CI): T3, -18.20 (−34.70, −1.70)](Fig. S2). However, when changing the region of interest to the lumbar spine, no significant results were found across all subgroups (Fig. S3).

### 3.4 Association of dietary carbohydrate intake with osteoporosis and fracture

**Fig. 5:**
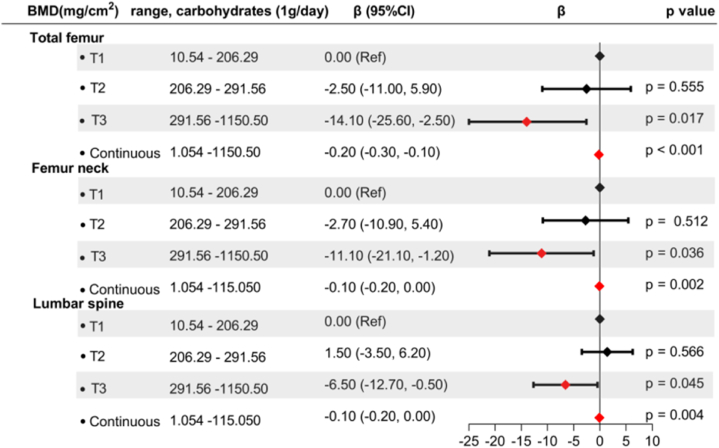
**Dietary carbohydrate intake level in fracture and non-fracture group**. **(a)** Dietary carbohydrate intake level by fracture status (Mann-Whitney *U* test). **(b)** Distribution of dietary carbohydrate intake by fracture status (Chi-square test).

Considering the significant association between carbohydrate intake and BMD, we proposed that varying levels of carbohydrate intake may also impact the risk of osteoporosis and fractures. The study analyzed the dietary carbohydrate intake in individuals with fractures at the hip, wrist, and spine. The results were consistent with those found in the osteoporosis group (Fig. 2a–b) and individuals with hip (p < 0.001) and spine (p < 0.001) fracture exhibited lower carbohydrate intake than those without fractures (Fig. 5a). On the contrary, participants with wrist fractures showed a higher carbohydrate intake than those without wrist fractures (p < 0.001). Further analysis indicated no significant differences in the distribution of carbohydrate intake between individuals with hip (p = 0.589) or spine fracture (p = 0.106) and the non-fracture group, while a marked increase of carbohydrate intake in the highest tertile was observed among those with wrist fractures (p = 0.003) (Fig. 5b).

**Fig. 6:**
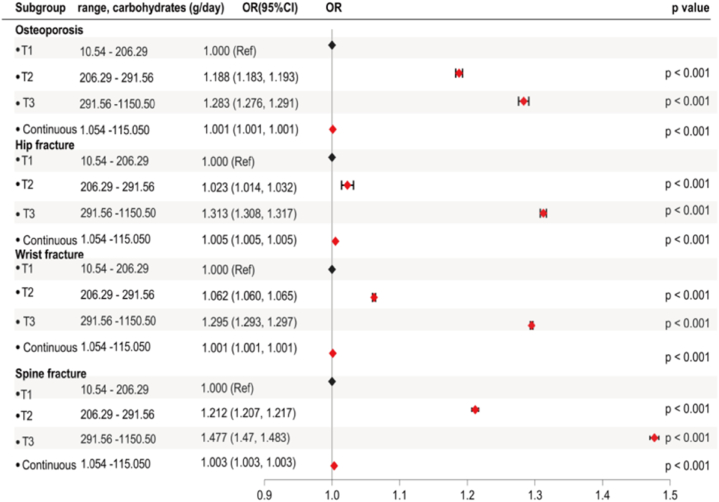
**Forest plot for the association of carbohydrate intake with the risk of osteoporosis and fracture at hip, wrist and lumbar spine from NHANES 2005**–**2010**. Weighted logistics analysis adjusted for age, gender, race/ethnicity, education levels, poverty income ratio, body mass index, smoking, physical activity level, alcohol consumption patterns, coronary heart disease, hypertension, albumin levels, total calcium, serum vitamin D, phosphorus, alkaline phosphatase, AST, ALT, triglycerides, total cholesterol, blood urea nitrogen, uric acid, serum glucose, bilirubin, total protein, arthritis, thyroid problem, stroke, the intake of calcium and total cholesterol. **Abbreviations**: CI, confidence interval; NHANES, National Health and Nutrition Examination Survey; BMD, bone mineral density; ALP, Alkaline phosphatase; ALT, Alanine aminotransferase; AST, Alanine aminotransferase; BUN, Blood urea nitrogen.

Based on the observed differences in carbohydrate intake across different groups, a weighted logistic regression was performed to analyze the association between carbohydrate and the risk of osteoporosis and fractures, using carbohydrate intake as both a continuous variable and categorical variable, respectively (Fig. 6). The results showed that an increase in carbohydrate intake, as a continuous variable, was associated with a slight increase in the risk of osteoporosis [OR (95%CI), p-value: 1.001 (1.001, 1.001), p < 0.001], as well as fractures at the hip [OR (95%CI), p-value: 1.005 (1.005, 1.005), p < 0.001], wrist [OR (95%CI), p-value: 1.001 (1.001, 1.001), p < 0.001], and spine [OR (95%CI), p-value: 1.003 (1.003, 1.003), p < 0.001]. These data indicate that an increase of 1g in dietary carbohydrate intake is associated with a 0.1 %–0.5 % increased risk of osteoporosis and fractures. When considering carbohydrate intake levels as a categorical variable and comparing them with the lowest tertile, the results showed that higher carbohydrate intake in the highest tertile was significantly associated with an increased risk of osteoporosis [T, OR (95%CI), p-value: T3, 1.283 (1.276, 1.291), p < 0.001], as well as fractures at the hip [T, OR (95%CI), p-value: T3, 1.313 (1.308, 1.317), p < 0.001], wrist [T, OR (95%CI), p-value: T3, 1.295 (1.293, 1.297), p < 0.001], and spine [T, OR (95%CI), p-value: T3, 1.477 (1.470, 1.483), p < 0.001]. Additionally, a trend test based on OR values, revealed a significant enhancement in the positive effect of carbohydrate intake on the risk of osteoporosis (p-_trend_ < 0.001) and fractures at the hip (p-_trend_ < 0.001), wrist (p-_trend_ < 0.001) and spine (p-_trend_ < 0.001) when compared to individuals with lowest levels of carbohydrate intake.

## 4 Discussion

In this large-scale population-based database of NHANES, the present study investigated comprehensively the association between dietary carbohydrate intake and BMD among participants without diabetes, and found regarding BMD in total femur, femoral neck and lumbar spine, dietary carbohydrate intake exerted a negative effect on BMD, and a higher level of carbohydrate consumption had a stronger effect. According to the results of subgroup analysis, the negative association of dietary carbohydrate intake with BMD was significantly different by age, sex and race, and participants aged 65–85 years, women, non-Hispanic whites tend to have a lower BMD in the total femur and femoral neck. Furthermore, we explored the effect of dietary carbohydrate intake on risk of osteoporosis and fractures, and confirmed that carbohydrate consumption might be positively associated with risk of osteoporosis and fractures, and a higher level of dietary carbohydrate consumption also had a stronger effect. These findings may be noteworthy for individuals with high carbohydrate intake, such as professional athletes and sports enthusiasts.

Bone remodeling is a dynamic physiological process that involves the coordination of both bone formation and resorption [33]. Genetic factors play a significant role in driving this dynamic process, with the remaining variance being explained by environmental factors, including lifestyle and dietary nutrition [5]. Multiple pieces of evidence suggest that various macronutrients, especially protein, fat and carbohydrate, influence both bone growth and bone loss [6,10,34]. Carbohydrate, along with proteins and fats, is one of the three major nutrients in the human diet, and complex carbohydrate structures such as disaccharides, oligosaccharides, and polysaccharides undergo digestion in the digestive system, breaking down into their simplest units including fructose, glucose and galactose. These simple units serve as a crucial source of energy in the human body but a high carbohydrate diet may be detrimental to skeletal health [35,36]. A cross-sectional study involving 3236 Scottish women age 50–59 years suggested that a high-carbohydrate diet could be a risk factor for osteoporosis [8]. It was found that the intake of processed foods and snack food high in carbohydrate were associated with a decrease in BMD. Similarly, another observational study from Brazil also demonstrated that excessive consumption of sweets foods exerted a negative effect on BMD, indicating sugar as a refined carbohydrate may have an inverse effect on bone remodeling [9]. A more recent observational study focusing on the effect of distribution of macronutrients on BMD also indicated that a low carbohydrate with high-protein diet intake would be beneficial for prevention of bone loss in adults, while the higher percentage of carbohydrate intake may be harmful to bone remodeling and contribute to a higher risk of bone loss [10]. Low bone mass in osteoporosis increases risk of fractures, but previous studies show that the association between carbohydrate intake and fracture risk is still controversial. Among these studies, meta-analyses studies failed to find a significant association between fractures and carbohydrate intake [[11], [12], [13], [14], [15]]; and two cohort studies conducted in the United States have indicated that higher carbohydrate consumption may lower the risk of fractures, particularly in hip and wrist [16,17]. Nonetheless, the results of the present study were supported by a matched case-control study conducted among elderly individuals in China. This study found an association between a prudent dietary pattern characterized by reduced intake of grains (primarily composed of carbohydrates) and a decreased risk of hip fractures, potentially indicating adverse effects of a high carbohydrate diet on fracture risk [37].

Compared with the present study, previous studies generally pay attention to carbohydrate as one component within diverse dietary patterns and have failed to highlight the direct impact of carbohydrate intake alone on bone density. Moreover, it is well established that serum glucose levels are positively associated with BMD in individuals with diabetes, and unlike general osteoporosis, patients with diabetes often exhibit normal to high BMD [[23], [24], [25]]. Therefore, it is crucial to consider the diabetic status when investigating the role of carbohydrate intake in bone metabolism. Unfortunately, previous studies rarely focused on these aspects. Additionally, inconsistent results regarding the association between fracture and carbohydrate from various studies may be due to differences in study design, such as inadequate control of confounding factors, particularly the lack of consideration for other nutrients like the intake of calcium and total cholesterol that can significantly influence the outcomes. To ensure the accuracy and reliability of the results, we comprehensively considered a variety of variables that affect bone metabolism. Moreover, the study further eliminated the individuals with diabetes and provided a better understanding on the association between carbohydrate intake alone and bone health in non-diabetic individuals. The results was consistent with published studies and showed increased dietary carbohydrate intake was significantly associated with low BMD [[8], [9], [10]] and high risk of fracture [37] among nondiabetic adults.

The gradual loss of bone tissue with advancing age is a natural process in the body, but this process can be accelerated by certain unfavorable factors including advanced age, females and non-Hispanic whites [38]. Females with declining estrogen levels have a much higher incidence of osteoporosis compared to males. According to a 2010 report on European Union (EU) indicated that while 5 million males were diagnosed with osteoporosis, there were 22 million females affected by the condition [38]. Furthermore, the incidence of osteoporosis and osteoporotic fractures increases with age, particularly affecting females over the age of 60 (60 %) and males over the age of 70 (20 %) in EU member states [38]. Existing evidence also suggests that differences in vitamin D and calcium metabolism, influenced by genetic factors, contribute to a higher prevalence of osteoporosis among non-Hispanic white individuals compared to other ethnicities [39]. The present study also found that factors such as age (65–85 years), gender (women), and ethnicity (non-Hispanic whites) intensified detrimental effect of high dietary carbohydrate intake (>291.56 g/day) on BMD.

The findings were also supported by previous research on the mechanisms underlying the negative impact of dietary carbohydrate intake on bone metabolism [40]. Several studies have identified that fructose, glucose, and galactose, which serve as fundamental components of carbohydrates, all can separately affect bone metabolism through various potential mechanisms. Glucose, as a common monosaccharide, provides energy for various metabolic activities. Almost all cellular activities in bone remodeling are strongly depended on glucose-mediated energy metabolism [41], so the change of glucose concentration may impair bone metabolism [42]. Bone marrow-derived mesenchymal stem cells (BMSCs) play a central role in bone homeostasis. However, a high-glucose microenvironment has been observed to activate Glycogen synthase kinase-3 (GSK-3), an enzyme widely expressed and known to reduce bone density. This activation inhibits the proliferation, migration, and osteogenic differentiation of BMSCs [43]. Furthermore, studies have shown that elevated glucose levels can severely impair the osteogenic function of osteoblasts through the disruption of mitochondrial energy metabolism, resulting in the accumulation of reactive oxygen species (ROS) and lipid peroxides that accelerate apoptosis and autophagy of osteoblasts [44]. In addition to BMSCs and osteoblasts, high glucose levels in diabetes also have been found to enhance osteoclast differentiation and increase bone resorption, ultimately leading to bone loss [42]. Moreover, osteocytes, which represent the terminal stage of osteoblast differentiation, play a role in regulating bone homeostasis by communicating with the surrounding environment. Elevated blood glucose adversely affects osteocytes by inducing iron-mediated cell death, resulting in long-term changes in bone cells and disrupted bone metabolism [45].

Fructose is another type of monosaccharide that occurs widely in nature and commonly found in fruits and some plants. Research findings indicate that elevated serum insulin levels in conjunction with oral carbohydrate intake can disturb calcium homeostasis. This disturbance manifests as reduced renal tubular reabsorption of calcium, leading to increased excretion of postprandial urinary calcium [46,47]. Among all carbohydrate types, fructose consumption exhibits the most significant effect on this phenomenon. As a response to high fructose intake, long-term depressed calcium may be detrimental to the balance between bone absorption and formation, particularly when dietary magnesium is low [48]. However, the detrimental effects induced by high carbohydrate intake could be attenuated through lowering carbohydrate and elevating composition of protein, dairy and calcium [49]. Relevant mechanisms are scarce, but merging research suggests that gut microbiota may be involved in the link between fructose intake and bone metabolism. Excessive fructose consumption has been shown to impact the quantity and diversity of bacterial species in the lumen of intestine; and this change can disrupt the normal tricarboxylic acid cycle of energy metabolism, potentially leading to aberrant bone metabolism characterized by diminished bone trabeculae and decreased bone mass [50].

Lactose, comprised of glucose and galactose, stands as the principal carbohydrate in dairy products. Currently, there is a widespread belief that a diet rich in dairy contributes to enhanced biological utilization of calcium from diverse sources during various developmental stages, fostering bone mineralization, growth, heightened density, and mitigated risk of osteoporotic fractures [[51], [52], [53], [54]]. However, this favorable effect appears to diminish post-adulthood [55,56], as most studies on calcium absorption in healthy adults suggest that dietary dairy products with varying lactose content have no significant impact on calcium absorption [57,58]. Furthermore, certain studies suggest that the ingestion of lactose from dairy might exert adverse effects on bone health. A Swedish cohort study uncovered an association between milk consumption and the risk of osteoporosis and fractures, possibly due to the elevated level of the oxidative stress marker, 8-iso-PGF2α, accompanying lactose intake, which correlates negatively with BMD [59]. Of greater significance, low BMD is a feature of the genetic metabolic disorder galactosemia [60]. In these patients, the enzyme involved in galactose metabolism, galactose-1-phosphate uridyltransferase (GALT) enzyme, is deficient, leading to the accumulation of galactose and galactose-1-phosphate, which may have detrimental effects on tissues, including bones [[61], [62], [63]]. These mechanisms may elucidate the adverse effects of lactose or galactose intake, yet further interventional studies are requisite for verification.

There were several limitations in the study. Due to the cross-sectional study design, it was theoretically not feasible to determine the causal relationship between carbohydrate intake and BMD, osteoporosis, and fractures. Additionally, the dietary data were averaged over two non-consecutive days, which may have ignored fluctuations in carbohydrate intake and affected the generalisability of the results. Furthermore, since carbohydrates are complex compounds, analyzing specific types of carbohydrates falls outside the scope of this study. For instance, the study failed to separately evaluate the impact of complex structures such as sucrose, cellulose, and starch, as the metabolic processes of these complex carbohydrates might also affect bone metabolism. Lastly, osteoporotic fractures are typically associated with low-energy impacts. However, our study did not exclude fractures resulting from high-energy traumatic mechanisms, recognizing that the influence of carbohydrate intake on fractures could vary based on the mechanism of injury. Therefore, prospective and well-designed studies are necessary to further evaluate the effects of carbohydrates on bone health.

## 5 Conclusion

In conclusion, the results from the current study indicated that a higher carbohydrate diet is associated with lower BMD and higher risk of osteoporosis and fractures among adults without diabetes. Moreover, a higher carbohydrate consumption in people aged 65 and over, women, and non-Hispanic whites show a stronger effect. Notably, these findings were observed in osteoporosis patients with lower total dietary energy intake and lower total intakes of protein, fat, and carbohydrates.

## Funding

This work was supported by the Chongqing Acute and Critical Care Clinical Medical Research Center (NO. 3354181), 10.13039/501100004829Science & Technology Department of Sichuan Province (No. 2021YFG0324) and 10.13039/501100020207Health Commission of Sichuan Province (No. 23LCYJ032).

## Data share statement

Publicly available datasets were analyzed in this study. The data can be found here: https://wwwn.cdc.gov/nchs/nhanes/default.aspx (accessed May 10, 2023).

## Ethics approval and consent to participate

The protocols of NHANES were approved by NCHS Ethics Review Board: https://www.cdc.gov/nchs/nhanes/irba98.htm).

NHANES has obtained written informed consent from all participants: https://www.cdc.gov/nchs/nhanes/genetics/genetic_participants.htm.

## Consent for publication

All authors approved the final version of the manuscript.

## Additional information

No additional information is available for this paper.

## Data availability statement

The data we used is available on the official website of the National Health and Nutrition Examination Survey (NHANES). (https://www.cdc.gov/nchs/nhanes/index.htm; NHANES Data 2005–2010).

## CRediT authorship contribution statement

**Ran Chen:** Writing – review & editing, Writing – original draft, Resources, Project administration, Methodology, Investigation, Formal analysis, Data curation, Conceptualization. **Kai Gong:** Writing – review & editing, Writing – original draft, Supervision, Software, Funding acquisition, Formal analysis, Data curation, Conceptualization. **Wei Chen:** Project administration, Formal analysis, Data curation. **Zongfeng Chen:** Formal analysis, Data curation. **Lianyang Zhang:** Funding acquisition, Data curation. **Ying Tang:** Writing – review & editing, Supervision, Investigation. **Yang Li:** Writing – review & editing, Software, Methodology, Investigation. **Siru Zhou:** Writing – review & editing, Conceptualization.

## Declaration of competing interest

The authors declare that they have no known competing financial interests or personal relationships that could have appeared to influence the work reported in this paper.

## Figures and Tables

**Table:** 

Abbreviations	Meaning
ALP	Alkaline phosphatase
ALT	Alanine aminotransferase
AST	Aspertate aminotransferase
BMD	Bone mineral density
BMI	Body mass index
BMSCs	Bone marrow-derived mesenchymal stem cells
BUN	Blood urea nitrogen
CDC	Centers for Disease Control and Prevention
CI	Confidence interval
DXA	Dual-energy X-ray absorptiometry
GSK-3	Glycogen synthase kinase-3
NCHS	National Center for Health Statistics
NHANES	National Health and Nutrition Survey
OR	Odd ratio
PIR	Poverty income ratio
ROS	Reactive oxygen species
