# Supplementary material for: Association of dietary carbohydrate intake with bone mineral density, osteoporosis and fractures among adults without diabetes: Evidence from National Health and Nutrition Examination Survey
Source: Heliyon. 2024 Aug 2;10(15):e35566. doi: 10.1016/j.heliyon.2024.e35566 (PMC11336747; doi:10.1016/j.heliyon.2024.e35566)
Supplement: Multimedia component 1 [file mmc1.docx]

*Human Participant Declaration Form (Heliyon)*

Title of the study involving human participant(s): **Association of dietary carbohydrate intake with bone mineral density, osteoporosis and fractures among adults without diabetes: Evidence from** **National Health and Nutrition Examination Survey**

Author Name(s): Ran Chen, Kai Gong, Wei Chen, Zongfeng Chen, Lianyang Zhang, Ying Tang, Yang Li, Siru Zhou

Institution(s):

1. War Trauma Medical Center, State Key Laboratory of Trauma and Chemical Poisoning, Army Medical Center, Daping Hospital, Army Medical University, Chongqing, 400042, P. R.China.
2. Department of Orthopaedics, The First Affiliated Hospital of Chengdu Medical College, Chengdu, Sichuan Province, 610500, P. R.China

Date: 2024.5.23

I, the undersigned author of the above-mentioned study, hereby declare the following:

1. I have obtained written informed consent from the participant(s) / patient(s) for the publication of this study, any accompanying data and images. Where consent was obtained from someone other than the participant(s) / patient(s), I confirm that this proxy was authorised to provide consent on the participant’s / patient’s behalf.
2. Where the participant(s) / patient(s) is/are a minor(s), we followed local laws on the age and circumstances under which they may consent for themselves. If they were not of legal age to consent, consent was obtained from an authorised proxy i.e., the parents or legal guardian(s). If the minor(s) has/have reasonable understanding of the informed consent and implications, signature (or assent, as appropriate) was also obtained from the minor(s).
3. Where the participant(s) / patient(s) provided consent themselves, I confirm that they had capacity to do so, and any mental or physical disabilities were taken into consideration in the process of informing and obtaining written informed consent.
4. Where the participant(s) / patient(s) has/have died, I confirm that the consent given still allows for publication.
5. I confirm that all content presented in this study, associated data and images have been deidentified and anonymized to the best possible extent.
6. The original signed and dated consent form is held by the treating institution or appropriate governing local / regional / national body and will be retained according to the policies and procedures of the institution or governing body.
7. The written informed consent form (please **do not** include with your submission) includes all relevant information pertinent to each participant / patient (such as the name, age, condition, medical history, diagnosis, and treatment)
8. The participant(s) / patient(s) / authorized proxy were fully informed of the purpose of this study, the potential risks and benefits of publication, and the consequence of disclosing their personal information.
9. The participant(s) / patient(s) or authorized proxy were given the opportunity to ask questions regarding publication of the study, had their questions answered fully and have consented to publish all associated data and images. In the case of clinical studies, the participant(s) / patient(s) or authorized proxy approved the final version of the manuscript.
10. The participant(s) / patient(s) or legal guardian(s) were informed that their consent and participation in the publication of this study is entirely voluntary and that they have the right to withdraw their consent at any time.
11. If this is a clinical study manuscript, I confirm that at least one of the authors of this paper was involved in the care of the participant(s) / patient(s).
12. I confirm that my article complies with the appropriate local / regional / national law on consent and privacy.

By signing this declaration form, I acknowledge that I have read and understood the information provided above, and I attest to the accuracy of this declaration. I understand that any false or misleading information may result in the rejection of the manuscript or other disciplinary actions.

As corresponding author, I hereby declare that I sign this document on behalf of all the authors of the above-mentioned study involving human participants.

| Corresponding author’s signature: | Siru Zhou |
| --- | --- |
| Date: | 2024.5.23 |

Please submit this **Human Participant Declaration Form** along with the manuscript to the journal. **Note:** The written informed consent form must NOT be submitted with your manuscript but must be made available to the journal if specifically requested.

Please retain a copy of this declaration for your records.

| Quantitative variables | | 11 | Explain how quantitative variables were handled in the analyses. If applicable, describe which groupings were chosen and why | Page 6 | | Line 150-155 |
| --- | --- | --- | --- | --- | --- | --- |
| Statistical methods | | 12 | (*a*) Describe all statistical methods, including those used to control for confounding | Page 7-8 | | Line 175-200 |
|  |  |  | (*b*) Describe any methods used to examine subgroups and interactions | Page 10 | | Line 263-264 |
|  |  |  | (*c*) Explain how missing data were addressed | Page 7-8 | | Line 198-200 |
|  |  |  | (*d*) *Cohort study*—If applicable, explain how loss to follow-up was addressed  *Case-control study*—If applicable, explain how matching of cases and controls was addressed  *Cross-sectional study*—If applicable, describe analytical methods taking account of sampling strategy | Page 7 | | Line 196 |
|  |  |  | (*e*) Describe any sensitivity analyses | N/A | | N/A |
| Results | | | | | | |
| Participants | | 13* | (a) Report numbers of individuals at each stage of study—eg numbers potentially eligible, examined for eligibility, confirmed eligible, included in the study, completing follow-up, and analysed | Page 8 | | Line 207 |
|  |  |  | (b) Give reasons for non-participation at each stage | N/A | | N/A |
|  |  |  | (c) Consider use of a flow diagram | Page 5 | | Line 113 |
| Descriptive data | | 14* | (a) Give characteristics of study participants (eg demographic, clinical, social) and information on exposures and potential confounders | Page 8 | | Line 207-216 |
|  |  |  | (b) Indicate number of participants with missing data for each variable of interest | Page 5 | | Line 112-118 |
|  |  |  | (c) *Cohort study*—Summarise follow-up time (eg, average and total amount) | N/A | | N/A |
| Outcome data | | 15* | *Cohort study*—Report numbers of outcome events or summary measures over time | N/A | | N/A |
|  |  |  | *Case-control study—*Report numbers in each exposure category, or summary measures of exposure | N/A | | N/A |
|  |  |  | *Cross-sectional study—*Report numbers of outcome events or summary measures | Page 8 | | Line 207 |
| Main results | | 16 | (*a*) Give unadjusted estimates and, if applicable, confounder-adjusted estimates and their precision (eg, 95% confidence interval). Make clear which confounders were adjusted for and why they were included | Page 6 | | Line 158-160 |
|  |  |  | (*b*) Report category boundaries when continuous variables were categorized | Page 6 | | Line 153-155 |
|  |  |  | (*c*) If relevant, consider translating estimates of relative risk into absolute risk for a meaningful time period | N/A | | N/A |
| Other analyses | | 17 | Report other analyses done—eg analyses of subgroups and interactions, and sensitivity analyses | Page 10 | | Line 261-273 |
| Discussion | | | | | | |
| Key results | 18 | | Summarise key results with reference to study objectives | Page 11 | Line 308-318 | |
| Limitations | 19 | | Discuss limitations of the study, taking into account sources of potential bias or imprecision. Discuss both direction and magnitude of any potential bias | Page 15 | Line 422-430 | |
| Interpretation | 20 | | Give a cautious overall interpretation of results considering objectives, limitations, multiplicity of analyses, results from similar studies, and other relevant evidence | Page 11-15 | Line 319-421 | |
| Generalisability | 21 | | Discuss the generalisability (external validity) of the study results | Page 15 | Line 424-426 | |
| Other information | | |  | | | |
| Funding | 22 | | Give the source of funding and the role of the funder for the present study and, if applicable, for the original study on which the present article is based | Page 19 | 531-533 | |

*Give information separately for cases and controls in case-control studies and, if applicable, for exposed and unexposed groups in cohort and cross-sectional studies.

**Note:** An Explanation and Elaboration article discusses each checklist item and gives methodological background and published examples of transparent reporting. The STROBE checklist is best used in conjunction with this article (freely available on the Web sites of PLoS Medicine at http://www.plosmedicine.org/, Annals of Internal Medicine at http://www.annals.org/, and Epidemiology at http://www.epidem.com/). Information on the STROBE Initiative is available at www.strobe-statement.org.
