# Supplementary material for: Association of dietary carbohydrate intake with bone mineral density, osteoporosis and fractures among adults without diabetes: Evidence from National Health and Nutrition Examination Survey
Source: Heliyon. 2024 Aug 2;10(15):e35566. doi: 10.1016/j.heliyon.2024.e35566 (PMC11336747; doi:10.1016/j.heliyon.2024.e35566)
Supplement: Multimedia component 2 [file mmc2.docx]

**Supplementary Table 1** Defined variables.

**Supplementary Table 2** Univariate Logistics regression analysis of variables for osteoporosis and fractures

**Supplementary Figure 1** Forest plot for the association of carbohydrate intake with bone mineral density of total femur stratified by age, sex, race, age and BMI.

**Supplementary Figure 2** Forest plot for the association of carbohydrate intake with bone mineral density of femoral neck stratified by age, sex, race, age and BMI.

**Supplementary Figure 3** Forest plot for the association of carbohydrate intake with bone mineral density of lumbar spine stratified by age, sex, race, age and BMI.

| **Supplementary Table 1. Defined variables.** | | |
| --- | --- | --- |
| ***Variable*** | **Classification** | Definition |
| ***Physical activity*** | **Low** | No activity is reported or some activity is reported but not enough to Moderate High and moderate categories. |
|  | **Mod-rate** | Either of the following three criteria:  (a) 3 or more days of vigorous-intensity of at least 20 minutes per day;  or(b) 5 or more days of moderate-intensity and/or walking of at least 30 minutes per day or(c) 5 or more days of any combination of walking, moderate-or vigorous-intensity activities accumulating at least 600 MET-minutes/week. |
|  | **High** | Participants have at least 3 days of vigorous-intensity activity and accumulate at least 1500 MET minutes per week or 7 or more days of walking, moderate or vigorous-intensity activities in any combination, and accumulate at least 3000 MET minutes per week. |
| ***Smoking*** | **Non- smoking** | Cotinine<0.011 ng/ml; |
|  | **Passive smoking** | 0.011 ng/ml<= Cotinine<10 ng/ml; |
|  | **Active smoking** | Cotinine>=10 ng/ml. |
| ***Alcohol intake*** | **Non- drinking** | Non- drinking was categorized as none (0 g/d); |
|  | **Moderate drinking** | Low to Moderate drinker (0.1 to 27.9 g/d for men and 0.1 to 13.9 g/d for women); |
|  | **Heavy drinking** | Heavy drinking (≥28 g/d for men and ≥14 g/d for women). |
| ***Hypertension*** | **No** | - |
|  | **Yes** | The average of three consecutive measurements in SBP and DBP is calculated. Hypertension is defined by mean SBP ≥ 140 mmHg or mean DBP ≥ 90 mmHg or antihypertensive medication user or physician diagnosis. |
| ***Coronary heat disease*** | **No** | - |
|  | **Yes** | A doctor’s diagnosis of coronary heart disease is defined as Coronary heat disease. |
| ***Diabetes*** | **No** | - |
|  | **Yes** | Diabetes through the participant’s fasting blood glucose ≥126 mg/dL or hemoglobin A1c ≥ 6.5% or random blood glucose ≥200 mg/dL, or being told by a doctor or health professional that you have diabetes or sugar diabetes or are taking insulin or hypoglycemic drugs. |
| ***Thyroid problem*** | **No** | - |
|  | **Yes** | A doctor’s diagnosis of thyroid problem is defined as thyroid problem. |
| ***BMI*** | **Normal** | ≤24.9 |
|  | **Overweight** | 25-29.9 |
|  | **Obese** | ≥30 |
| ***PIR*** | **Low income** | PIR < 1 |
|  | **Middle income** | PIR 1-4 |
|  | **High income** | PIR > 4 |
| ***Arthritis*** | **No** | - |
|  | **Yes** | A doctor’s diagnosis of osteoarthritis or rheumatoid arthritis is defined as arthritis. |
| ***Stoke*** | **No** | - |
|  | **Yes** | A doctor’s diagnosis of stroke is defined as Stroke. |
| ***Osteoporosis*** | **No** | - |
|  | **Yes** | A doctor’s diagnosis of osteoporosis or T scores ≤ − 2.5 or take anti-osteoporosis is defined as osteoporosis |

**Abbreviation:** METs, metabolic equivalents, PIR, poverty income ratio, BMI, body mass index. Lumbar spine, femoral neck and total hip BMD were converted into T-scores using the formula: T-score= (BMDrespondent − mean BMD reference group)/SDreferencegroup.

| **Supplementary Table 2. Univariate Logistics regression analysis of variables for osteoporosis and fractures** | | | | | | | | | | | | |
| --- | --- | --- | --- | --- | --- | --- | --- | --- | --- | --- | --- | --- |
| **Variables** | **Univariate Logistics regression analysis** | | | | | | | | | | | |
|  | **Osteoporosis** | | | **Hip fracture** | | | **Wrist fracture** | | | **Spine fracture** | | |
|  | **OR** | **95%CI** | **P-value** | **OR** | **95%CI** | **P-value** | **OR** | **95%CI** | **P-value** | **OR** | **95%CI** | **P-value** |
| Age (years) | 1.092 | 1.092-1.092 | *** | 1.036 | 1.036-1.036 | *** | 0.997 | 0.996-0.997 | *** | 1.023 | 1.023-1.023 | *** |
| Gender | 12.99 | 12.94-13.04 | *** | 0.990 | 0.986-0.994 | *** | 0.767 | 0.767-0.768 | *** | 0.713 | 0.711-0.715 | *** |
| Race/ethnicity | 0.717 | 0.716-0.718 | *** | 0.669 | 0.667-0.670 | *** | 0.728 | 0.727-0.728 | *** | 0.649 | 0.648-0.650 | *** |
| Education levels | 0.710 | 0.709-0.711 | *** | 0.521 | 0.520-0.522 | *** | 1.011 | 1.010-1.012 | *** | 0.727 | 0.726-0.728 | *** |
| PIR | 0.861 | 0.860-0.862 | *** | 0.848 | 0.846-0.850 | *** | 0.971 | 0.970-0.972 | *** | 0.764 | 0.763-0.766 | *** |
| BMI | 0.776 | 0.775-0.777 | *** | 0.723 | 0.721-0.725 | *** | 1.037 | 1.037-1.038 | *** | 1.211 | 1.209-1.213 | *** |
| Waist circumference (cm) | 0.987 | 0.987-0.987 | *** | 0.996 | 0.995-0.996 | *** | 1.005 | 1.005-1.005 | *** | 1.022 | 1.022-1.022 | *** |
| Alcohol drinking | 0.557 | 0.557-0.556 | *** | 0.887 | 0.885-0.890 | *** | 1.126 | 1.125-1.127 | *** | 1.559 | 1.556-1.563 | *** |
| Physical activity | 0.899 | 0.899-0.900 | *** | 0.884 | 0.883-0.886 | *** | 1.094 | 1.094-1.095 | *** | 1.296 | 1.295-1.297 | *** |
| Smoking | 0.767 | 0.766-0.768 | *** | 1.582 | 1.577-1.586 | *** | 1.265 | 1.264-1.266 | *** | 1.716 | 1.712-1.719 | *** |
| Hypertension | 2.147 | 2.143-2.151 | *** | 0.937 | 0.933-0.941 | *** | 0.955 | 0.954-0.956 | *** | 0.936 | 0.933-0.939 | *** |
| Arthritis | 0.131 | 0.131-0.131 | *** | 0.217 | 0.217-0.218 | *** | 0.660 | 0.659-0.661 | *** | 0.278 | 0.278-0.279 | *** |
| Coronary heat disease | 0.315 | 0.313-0.317 | *** | 0.214 | 0.212-0.216 | *** | 1.204 | 1.198-1.210 | *** | 0.625 | 0.619-0.630 | *** |
| Stroke | 0.164 | 0.163-0.165 | *** | 0.223 | 0.222-0.225 | *** | 0.736 | 0.733-0.740 | *** | 0.226 | 0.225-0.228 | *** |
| Albumin (g/dL) | 0.880 | 0.880-0.881 | *** | 0.936 | 0.935-0.936 | *** | 1.018 | 1.018-1.019 | *** | 0.989 | 0.989-0.990 | *** |
| Total calcium (mmol/L) | 3.818 | 3.776-3.860 | *** | 0.050 | 0.049-0.051 | *** | 2.397 | 2.381-2.413 | *** | 0.657 | 0.647-0.667 | *** |
| Vitamin D (nmol/L) | 1.006 | 1.006-1.006 | *** | 1.008 | 1.008-1.008 | *** | 1.003 | 1.003-1.003 | *** | 1.004 | 1.004-1.004 | *** |
| ALP (U/L) | 1.008 | 1.008-1.008 | *** | 1.012 | 1.012-1.012 | *** | 1.007 | 1.007-1.007 | *** | 1.011 | 1.011-1.011 | *** |
| Phosphorus (mmol/L) | 2.965 | 2.949-2.981 | *** | 4.122 | 4.082-4.161 | *** | 1.047 | 1.043-1.050 | *** | 0.802 | 0.797-0.808 | *** |
| Total cholesterol (mmol/L) | 1.152 | 1.151-1.153 | *** | 0.747 | 0.746-0.749 | *** | 1.121 | 1.121-1.122 | *** | 1.375 | 1.374-1.377 | *** |
| Triglycerides(mmol/L) | 0.986 | 0.985-0.986 | *** | 0.819 | 0.818-0.821 | *** | 1.043 | 1.043-1.043 | *** | 1.126 | 1.125-1.126 | *** |
| BUN (mmol/L) | 1.228 | 1.227-1.229 | *** | 0.945 | 0.944-0.946 | *** | 0.962 | 0.961-0.962 | *** | 0.886 | 0.885-0.886 | *** |
| Uric acid ( mmol/L) | 0.993 | 0.993-0.993 | *** | 1.000 | 1.000-1.000 | *** | 1.001 | 1.001-1.001 | *** | 1.001 | 1.001-1.001 | *** |
| ALT | 0.983 | 0.983-0.983 | *** | 1.008 | 1.007-1.008 | *** | 0.999 | 0.999-0.999 | *** | 0.998 | 0.998-0.998 | *** |
| AST | 1.001 | 1.001-1.001 | *** | 1.005 | 1.005-1.005 | *** | 1.000 | 1.000-1.000 | *** | 0.987 | 0.987-0.987 | *** |
| Bilirubin (mg/dL) | 0.262 | 0.261-0.263 | *** | 0.621 | 0.616-0.625 | *** | 1.113 | 1.111-1.115 | *** | 1.041 | 1.036-1.045 | *** |
| Calcium intake (mg) | 0.999 | 0.999-0.999 | *** | 1.000 | 1.000-1.000 | *** | 1.000 | 1.000-1.000 | *** | 1.000 | 1.000-1.000 | *** |
| Phosphorus intake (mg) | 0.999 | 0.999-0.999 | *** | 1.000 | 1.000-1.000 | *** | 1.000 | 1.000-1.000 | *** | 1.000 | 1.000-1.000 | *** |
| Total cholesterol intake (mg) | 0.998 | 0.998-0.998 | *** | 1.000 | 1.000-1.000 | *** | 1.000 | 1.000-1.000 | *** | 1.000 | 1.000-1.000 | *** |
| Total energy intake (Kcal) | 0.999 | 0.999-0.999 | *** | 1.000 | 1.000-1.000 | *** | 1.000 | 1.000-1.000 | *** | 1.000 | 1.000-1.000 | *** |
| Fat intake (g) | 0.986 | 0.986-0.986 | *** | 1.000 | 1.000-1.000 | *** | 1.002 | 1.002-1.002 | *** | 0.999 | 0.999-0.999 | *** |
| Protein intake (g) | 0.981 | 0.981-0.981 | *** | 0.998 | 0.998-0.998 | *** | 1.001 | 1.001-1.001 | *** | 0.996 | 0.996-0.996 | *** |

**Abbreviation:** BMI, body mass index; BMD, bone mineral density; ALP, Alkaline phosphatase; ALT, Alanine aminotransferase; AST, Alanine aminotransferase; BUN, Blood urea nitrogen. PIR, poverty income ratio; ***:<0.001


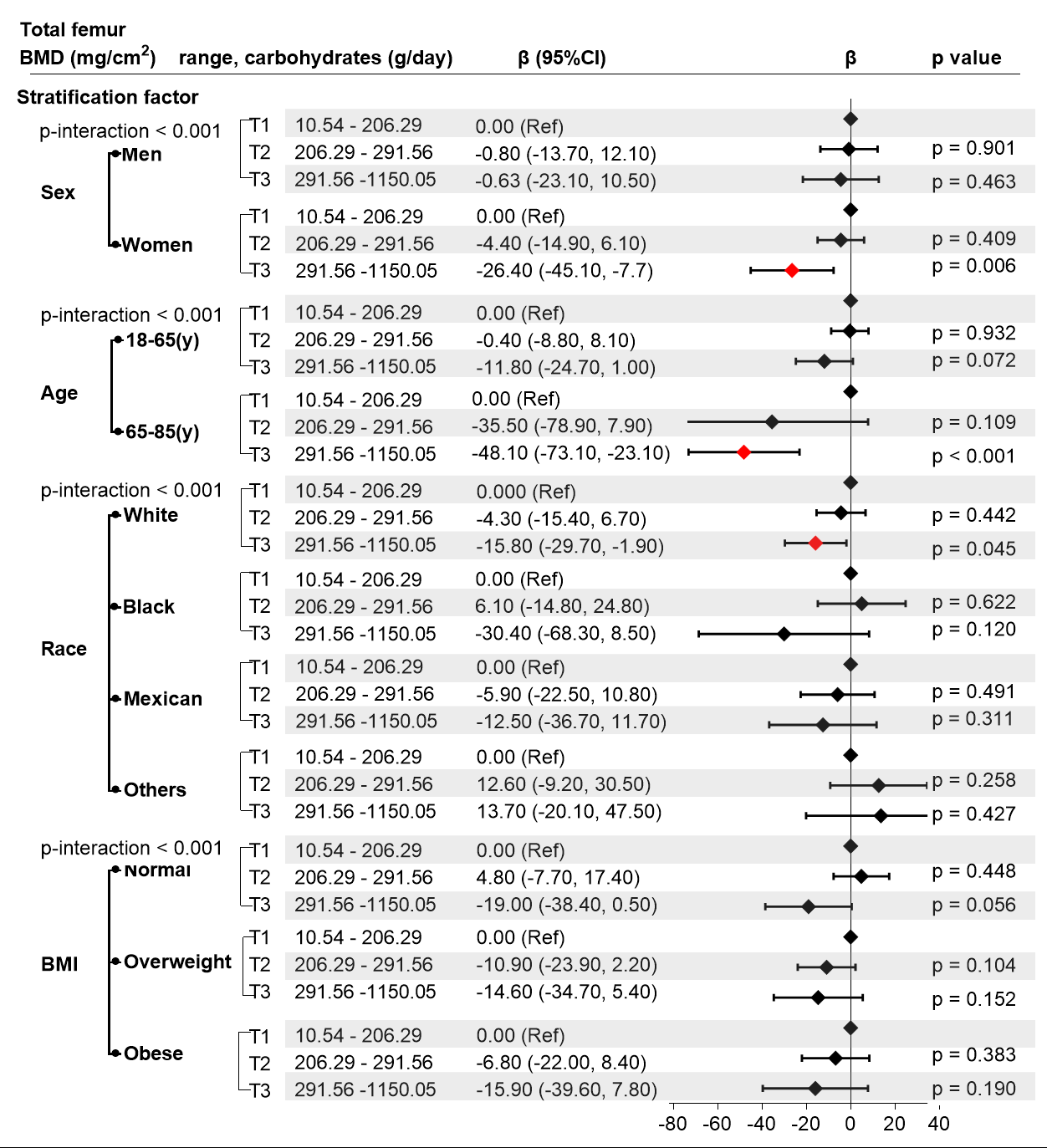


**Supplementary Figure 1 Forest plot for the association of carbohydrate intake with bone mineral density of total femur stratified by age, sex, race, age and BMI**. Weighted multiple linear regression adjusted for age, gender, race/ethnicity, education levels, poverty income ratio, body mass index, smoking, physical activity level, alcohol consumption patterns, coronary heart disease, hypertension, albumin levels, total calcium, serum vitamin D, phosphorus, alkaline phosphatase, AST, ALT, triglycerides, total cholesterol, blood urea nitrogen, uric acid, serum glucose, bilirubin, total protein, arthritis, thyroid problem, stroke, the intake of calcium and total cholesterol. **Abbreviations:** CI, confidence interval; NHANES, National Health and Nutrition Examination Survey; BMD, bone mineral density; ALP, Alkaline phosphatase; ALT, Alanine aminotransferase; AST, Alanine aminotransferase; BUN, Blood urea nitrogen.


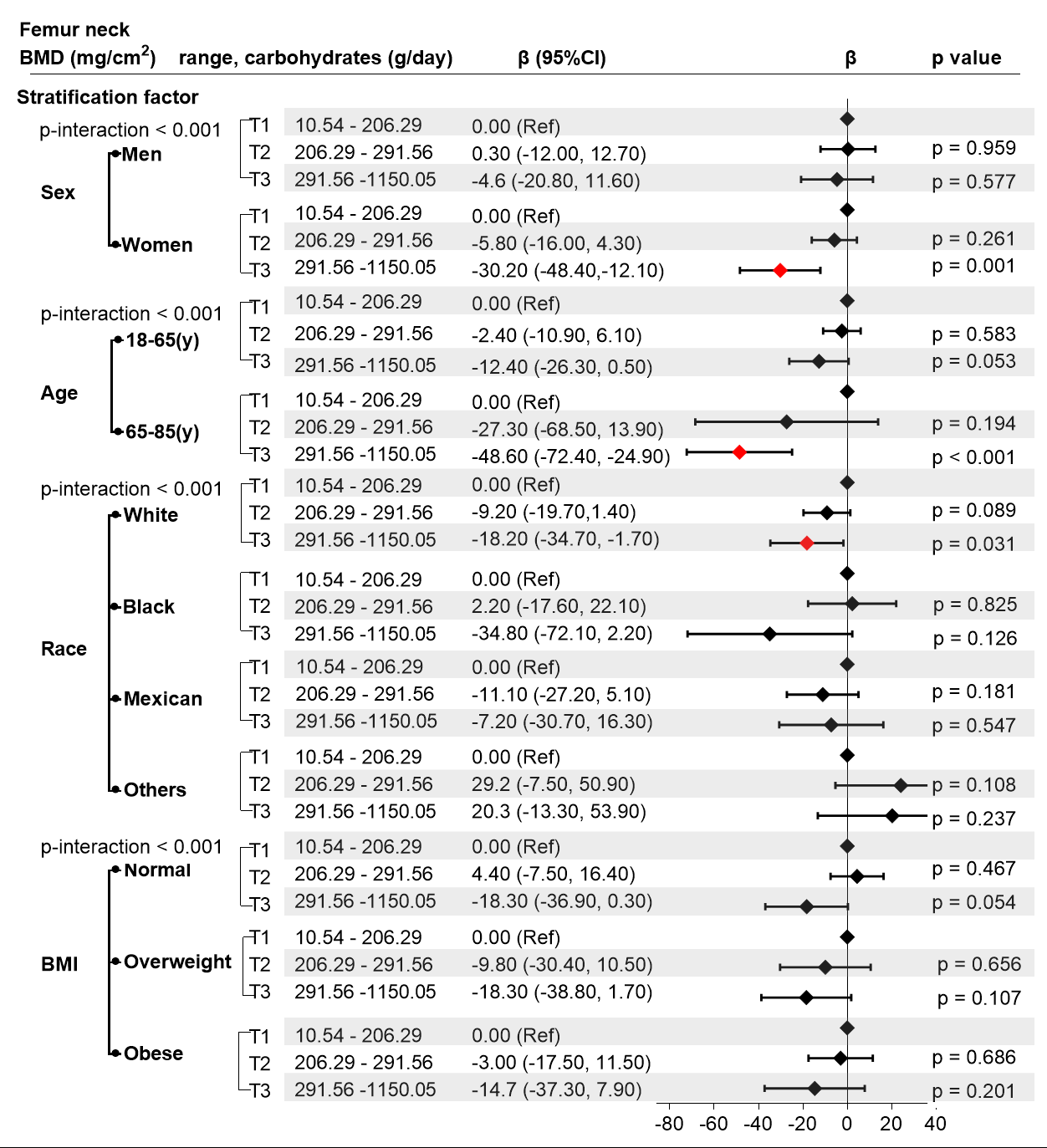


**Supplementary Figure 2 Forest plot for the association of carbohydrate intake with bone mineral density of femoral neck stratified by age, sex, race, age and BMI**. Weighted multiple linear regression adjusted for age, gender, race/ethnicity, education levels, poverty income ratio, body mass index, smoking, physical activity level, alcohol consumption patterns, coronary heart disease, hypertension, albumin levels, total calcium, serum vitamin D, phosphorus, alkaline phosphatase, AST, ALT, triglycerides, total cholesterol, blood urea nitrogen, uric acid, serum glucose, bilirubin, total protein, arthritis, thyroid problem, stroke, the intake of calcium and total cholesterol. **Abbreviations:** CI, confidence interval; NHANES, National Health and Nutrition Examination Survey; BMD, bone mineral density; ALP, Alkaline phosphatase; ALT, Alanine aminotransferase; AST, Alanine aminotransferase; BUN, Blood urea nitrogen.


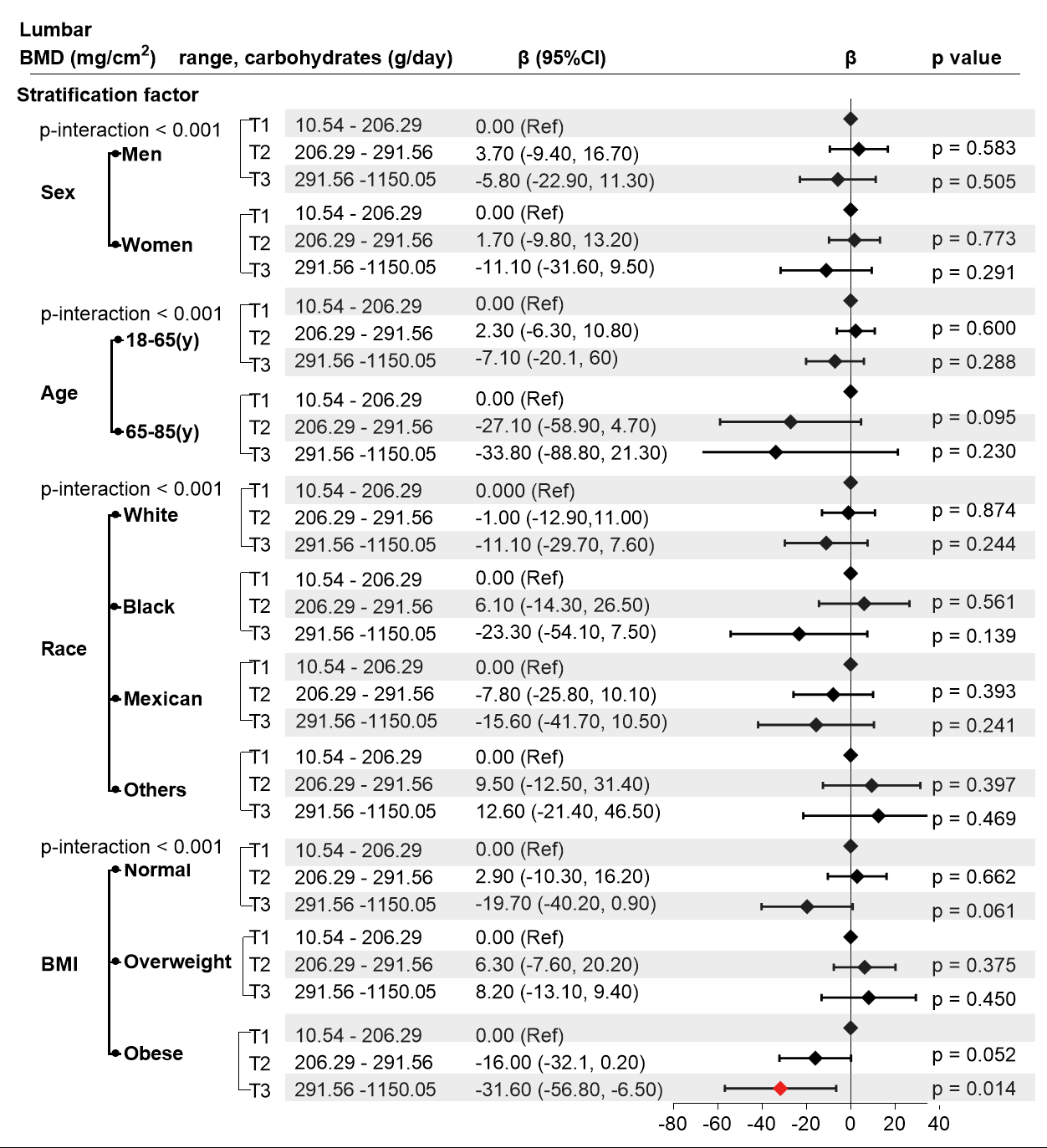


**Supplementary Figure 3 Forest plot for the association of carbohydrate intake with bone mineral density of lumbar spine stratified by age, sex, race, age and BMI**. Weighted multiple linear regression adjusted for age, gender, race/ethnicity, education levels, poverty income ratio, body mass index, smoking, physical activity level, alcohol consumption patterns, coronary heart disease, hypertension, albumin levels, total calcium, serum vitamin D, phosphorus, alkaline phosphatase, AST, ALT, triglycerides, total cholesterol, blood urea nitrogen, uric acid, serum glucose, bilirubin, total protein, arthritis, thyroid problem, stroke, the intake of calcium and total cholesterol. **Abbreviations**: CI, confidence interval; NHANES, National Health and Nutrition Examination Survey; BMD, bone mineral density; ALP, Alkaline phosphatase; ALT, Alanine aminotransferase; AST, Alanine aminotransferase; BUN, Blood urea nitrogen.
